# Multiple Painless Masses in a Peritoneal Dialysis Patient

**DOI:** 10.34067/KID.0000000000000085

**Published:** 2023-05-25

**Authors:** Wannasit Wathanavasin, Patchara Tanateerapong, Paweena Susantitaphong

**Affiliations:** 1 Nephrology Unit, Department of Medicine, Charoenkrung Pracharak Hospital, Bangkok Metropolitan Administration, Bangkok, Thailand; 2 Division of Nephrology, Department of Medicine, Faculty of Medicine, Chulalongkorn University, Bangkok, Thailand; 3 Research Unit for Metabolic Bone Disease in CKD Patients, Faculty of Medicine, Chulalongkorn University, Bangkok, Thailand

**Keywords:** tumoral calcinosis, peritoneal dialysis, hyperparathyroidism, hyperphosphatemia, extraosseous calcification

## Abstract

**Figure:**
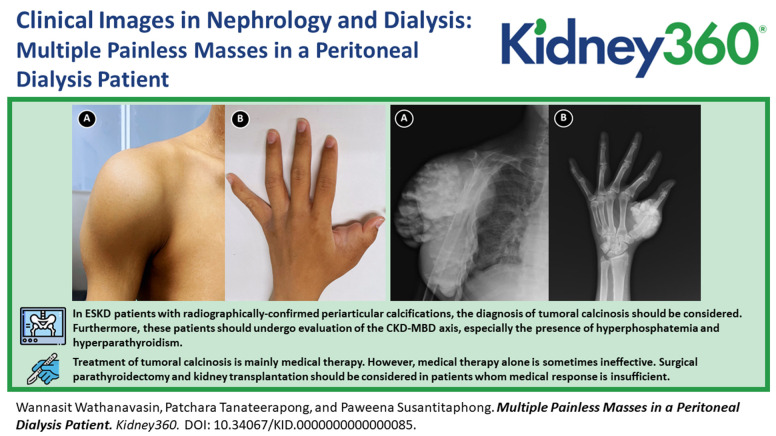

## Case Description

**Figure 1:**
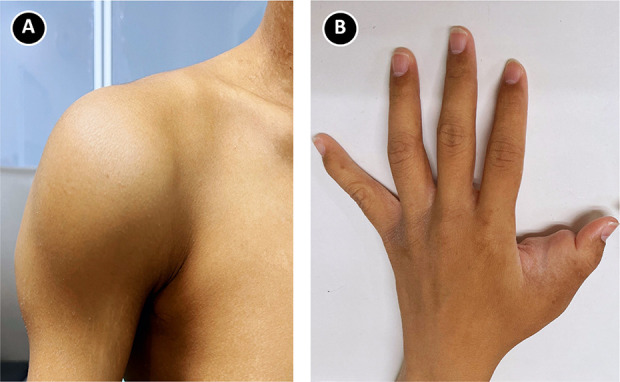
**Clinical photograph of the patient.** (A) Physical examination revealed a subcutaneous mass around right shoulder. (B) Physical examination revealed a subcutaneous mass of the proximal phalanx of the first finger of the left hand.

A 26-year-old man with anuric ESKD, secondary to chronic glomerulonephritis, undergoing peritoneal dialysis (PD) for 4 years, described progressively worsening swelling of his right shoulder and left first finger. His PD prescription comprised four exchanges of 2.0 L with standard calcium peritoneal dialysate (1.75 mmol/L). His peritoneal membrane transport was classified as high, on the basis of a dialysate-to-plasma ratio of creatinine (D/P_Cr_) of 0.82. His last weekly total KT/V urea and weekly creatinine clearance were 1.82 and 51.4 L, respectively. He began to complain of progressive painless palpable masses on his right shoulder and left hand for 1 year. On physical examination, stony hard consistency subcutaneous masses around the right shoulder (Figure 1A) and proximal phalanx of the first finger of the left hand (Figure 1B), measuring 18×15 cm and 6×5 cm, respectively, were noted. The range of motion of the right shoulder joint was significantly limited.

**Figure 2:**
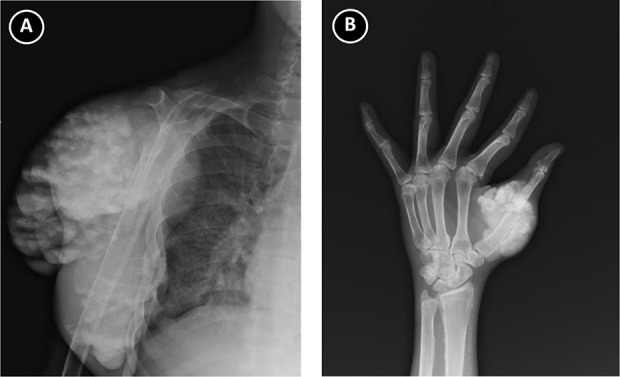
**Plain radiograph of the patient.** (A) Radiograph of right shoulder showed amorphous, multilobulated, and cystic calfication in periarticular location. (B) Radiograph of left hand showed amorphous, multilobulated calcification around promixal interphalangeal joints of the first finger.

Pertinent laboratory studies revealed slightly elevated serum calcium (Ca, 10.3 mg/dl) and a marked increase in serum phosphorus (P, 9.8 mg/dl) with elevated intact parathyroid hormone (PTH) levels (immunoreactive PTH, 1440 pg/ml). Plain radiographs of the right shoulder and left hand showed well-defined multilobulated (cloud-like) calcified masses (Figure 2, A and B), which were compatible with tumoral calninosis. A ^99^Tc-sestamibi scan revealed hyperplasia of the four parathyroid glands.

On the basis of his laboratory abnormalities of mineral and bone disorder (MBD) along with the typical characteristic radiographic findings, he was diagnosed with uremic tumoral calcinosis suspected from tertiary hyperparathyroidism. Standard calcium dialysate was replaced by low calcium dialysate (1.25 mmol/L) to avoid hypercalcemia. Dietary phosphate restriction and non–calcium-based phosphate binder were prescribed for correcting hyperphosphatemia. He was soon scheduled for surgical parathyroidectomy and was on the waiting list for kidney transplantation as the etiological therapeutic management.

## Discussion

Tumoral calcinosis is a rare form of the extraosseous calcification in patients with advanced CKD, characterized by massive deposition of calcium-phosphorus crystals in periarticular areas.^1^ Several CKD-MBD–related risk factors as demonstrated in this patient, including hypercalcemia, hyperphosphatemia, and an elevated calcium × phosphate product with coexistent severe hyperparathyroidism, have been implicated in the pathogenesis of this condition.^2^ Medical history together with radiological evaluation plays a key role in the diagnosis of tumoral calcinosis.^3^

This case demonstrated the typical appearance of tumoral calcinosis in plain radiographs, including amorphous, multilobulated, and cystic calcifications in a periarticular location of the shoulder and hand, the commonly affected sites. Treatment of tumoral calcinosis is mainly optimal control of the CKD-MBD aspect by medical therapy, including dietary phosphorus restriction, non–calcium-based phosphate binder, calcimimetics, and adequate dialysis with low-Ca dialysate. For persistent or refractory tumoral calcinosis, surgical parathyroidectomy and kidney transplantation are a good choice, which could lead to rapid resolution of lesions in some patients.^4,5^

## Teaching Points

- In all ESKD patients with radiographically confirmed periarticular calcifications, the diagnosis of tumoral calcinosis should be considered. Furthermore, these patients are recommended to evaluate the CKD-MBD aspect, especially the presence of hyperphosphatemia and hyperparathyroidism.
- Treatment of tumoral calcinosis is mainly medical therapy. However, medical therapy alone is sometimes ineffective. Surgical parathyroidectomy and kidney transplantation should be considered in patients in whom medical response is insufficient.
